# What is “moral distress” in nursing? How, can and should we respond to it?

**DOI:** 10.1111/jocn.14332

**Published:** 2018-04-02

**Authors:** Georgina Morley

**Affiliations:** ^1^ Centre for Ethics in Medicine Population Health Sciences University of Bristol Bristol UK; ^2^ Barts Heart Centre Barts Health NHS Trust West Smithfield London UK

Interest in moral distress (MD) as a research topic has soared in recent years. What is it about this concept that makes it so intriguing? Why does it create such debate amongst healthcare professionals? Why has there been so much conceptual confusion regarding the concept? And why do nurses, in particular, seem to feel this concept so accurately captures their experiences? These are some of the questions that I have been thinking about over the course of my doctoral studies and I would like to consider here.

As it was originally conceived, MD was believed to arise “when one knows the right thing to do, but institutional constraints make it nearly impossible to pursue the right course of action” (Jameton, 1984, p. 6). On this understanding, all that is required for MD to occur is a moral judgement and the presence of an external constraint which prevents that judgement from being carried out. This notion that nurses are constrained by external forces and are “not free to be moral” (Yarling & McElmurry, 1986, p. 63) has according to critics, such as Paley (2004), perpetuated a favourite metanarrative of nurses suffering within nursing discourse. Others, such as Johnstone and Hutchinson (2015), argue that the entire concept ought to be abandoned because it undermines the process of moral deliberation by perpetuating the notion that nurses’ moral judgements are correct and justified. They argue that MD, as it is currently understood (according to Jameton (1984, 1993)), risks nurses failing to nurture the skills required for ethical discussion and damages their integration into moral decision‐making because of the “assumed rightness of [their] moral judgements” (Johnstone and Hutchinson, 2015). Indeed, Weinberg (2009) highlights how Jameton's conception of MD fails to acknowledge the possibility that there might not even be a “correct” course of action. So, does Jameton's (1984) conception of MD perpetuate epistemic arrogance, or worse perhaps, epistemic laziness, when it comes to decision‐making? Should we, as Johnstone and Hutchinson (2015) argue, abandon the concept of MD altogether?

Despite criticism of the concept, the notion of MD continues to attract research attention from healthcare professionals and, in particular, nurses, suggesting that it appeals to individuals lived experiences. I was first drawn to exploration of this phenomenon through my own experiences working as a nurse in the National Health Service (NHS). I was surprised to find a distinct lack of empirical research had been conducted in the United Kingdom (UK). Since Jameton (1984) first defined MD, hundreds of qualitative, quantitative and theoretical studies exploring MD have been published, but very few are from the UK. In a recent review of this literature, Morley et al. (2017) identified twenty key definitions from the extensive MD literature, each made up of various necessary and/or sufficient conditions required for MD to occur (Morley, Ives, Bradbury‐Jones, & Irvine, 2017). However, a lack of engagement within the empirical literature with these various suggested definitions of MD has caused conceptual confusion which has complicated attempts to study the phenomenon. For example, there are many studies which purport to measure MD using variations of the Moral Distress Scale, one of the most commonly used measures of MD, but many of these studies use different definitions of MD to underpin their research which makes comparisons between studies problematic. The first aim of my research project was therefore to establish a definition of MD that was plausible within the UK setting. As with other recent theoretical efforts, I argue that Jameton's (1984) original definition of MD needs to be broadened and postulated a definition that comprises three core criteria:

1. the experience of a moral event,
2. the experience of “psychological distress”, and
3. a direct causal relation between (1) and (2).

This much broader understanding of MD allows for other potentially relevant causes of MD to be captured within the “umbrella” term of MD (McCarthy & Deady, 2008), which can then be further subcategorised, as suggested by Fourie (2015), into, for example, “moral‐constraint distress” or “moral‐conflict distress.” I hypothesize that broadening the definition of MD and subcategorising into these constituent pieces will allow for more specific measurements and targeted interventions to help address MD.

Arguably, only once the conceptual fog has cleared can healthcare professionals, researchers and policy‐makers can begin to gain further clarity regarding what, if anything, can be done to mitigate MD? Indeed, it has been suggested that MD is simply a natural response to morally troubling experiences that should be welcomed and that getting rid of MD is not only impossible but undesirable (personal communication with Tigard; hinted at in Tigard (2017) and Howe (2017)). MD may be a natural consequence of the messiness of moral life but when one experiences MD everyday due to their occupation, which seems to be the case for nurses and other healthcare professionals, then it may instead be regarded as an occupational hazard that employers have a responsibility to address. Marshall and Epstein (2016) argue that MD is inherently linked to the notion of “moral hazard” because of the power differentials between those making the decisions and those that must live with those decisions. Moral hazard is a term used to describe situations in which one party controls decisions about resources but another party bears the burden of those decisions (Brunnquell & Michaelson, 2016). Many nurses are in the position of bearing responsibility for enacting the decisions of their medical colleagues and seem most likely to risk experiencing the moral hazard that is MD. We need, therefore, to pay more attention to nurse's working environments and their potentially negative ramifications. The links between MD and the work environment are becoming increasingly well documented. Much of the empirical research exploring MD in North America has cautioned that higher levels of MD are often correlated with poorer perceived ethical climate (Hamric & Blackhall, 2007; Hamric, Borchers, & Epstein, 2012; Silén, Svantesson, Kjellström, Sidenvall, & Christensson, 2011) and intention to leave the workplace (Corley, Minick, Elswick, & Jacobs, 2005; Whitehead, Herbertson, Hamric, Epstein, & Fisher, 2014) Additionally, MD appears to be correlated with other experiences that have similar affects such as compassion fatigue and burnout (Maiden, Georges, & Connelly, 2011; Rushton, Batcheller, Schroeder, & Donohue, 2015).

Even if we perceive MD to be a natural response to morally challenging events, the negative consequences that MD appears to have upon the nursing profession (perpetuating the nursing shortage as increasing numbers of nurses leave the profession, psychological distress and distancing oneself from patients) suggest we should consider ways to respond to it. MD seems to consist of two fundamental aspects: psychological distress and a moral event. We might, therefore, need different interventions to tackle each of these aspects. There are several mechanisms in place that can be further utilized and integrated into everyday clinical practice that may help address psychological distress, for example regular team debriefing sessions after patients' deaths or clinical incidents, and addressing the stigma around using staff support services such as employee assistance programmes that are available at most NHS Trusts. In a recent longitudinal study, Maben et al. (2018) explored the effectiveness of Schwartz Rounds^®^ which offer a safe space for staff to come together and reflect upon the experiences and challenges they face at work. They found that attending Schwartz Rounds^®^ resulted in a statistically significant improvement in staff psychological well‐being, increased empathy and compassion for patients and colleagues, and positive changes in practice (Maben et al., 2018).

For other possible responses to MD, we can look to North America, where much of the initial MD research has been conducted and initial interventions tested. For example, Cynda Rushton, Professor of Clinical Ethics, Johns Hopkins University, has developed a Mindful Ethical Practice and Resilience Academy (MEPRA) based upon her work exploring moral resilience as a possible response to MD (Rushton, Caldwell, & Kurtz, 2016). Elsewhere in the USA, Helft, Bledsoe,Hancock, and Wocial (2009) have explored facilitated ethics conversations as a response to MD and Hamric & Epstein (2017) have launched a systemwide MD consultation service. In the UK, Traynor (2017) has explored the concept of “critical resilience” not as a specific response to MD but rather as a way for nurses to look more critically at their environments. Traynor (2017) argues that by examining and revealing the power structures in which we work, nurses can come together in solidarity and resist the external forces that Morley and Jackson (2017) suggest are destroying the art of nursing. Importantly, there is much more work to do regarding ways to support clinical staff regarding ways to support clinical staff facing not only the increasing external pressures but the complex everyday clinical ethical issues, and this is the future of my own work. However, it is important that we try to transform the nursing narrative, away from the metanarrative of powerless, suffering victims (Paley, 2004), and embrace a new narrative for nursing.

In a recent study reported in Peden‐McAlpine, Liaschenko, Traudt, and Gilmore‐Szott (2015) and Traudt, Liaschenko, and Peden‐McAlpine (2016), 19 experienced critical care nurses were interviewed who self‐identified as skilled and comfortable during end‐of‐life care, MD did not arise as a theme. This was surprising considering that many studies have found that end‐of‐life care and withdrawal of life‐sustaining treatments provides the kind of moral events that often lead to experiences of MD. Instead of discussing their experiences of MD, the nurses in Traudt et al. (2016), who had an average of 17 years critical care experience, reported feeling a strong sense of moral agency, felt accountable for their actions, possessed “moral imagination” (meaning they could empathise and appreciate the values of others) and perceived a “moral community” in which they viewed themselves as an integral part of the decision‐making process. The authors highlighted how the nurses in this study seemed to feel able to navigate ethically difficult scenarios. Commenting on this study, Rushton and Carse (2016) applaud the changing MD narrative, away from the powerlessness nurse to one in which the nurse is able to thrive within a moral community, bolstered by ethical competency, likely authority and able to enact their moral agency. Whilst we continue to implement, measure and assess the effectiveness of interventions that might mitigate the negative consequences of MD, I call on nurses to continue the fight against this continued narrative of powerlessness and instead embrace the power they do have to engage in moral reflection and debate.

## CONFLICT OF INTEREST

No conflict of interest.

## FUNDING INFORMATION

This work forms part of Morley's doctoral work which is funded by a Society and Ethics Research Fellowship for Healthcare Professionals from the Wellcome Trust (Grant Ref: 108640/Z/15/Z). The project is supervised by Dr Jonathan Ives (University of Bristol), Dr Caroline Bradbury‐Jones (University of Birmingham) and Professor Richard Huxtable (University of Bristol).
